# Immediate and sustained terminal complement inhibition with ravulizumab in patients with anti-aquaporin-4 antibody-positive neuromyelitis optica spectrum disorder

**DOI:** 10.3389/fneur.2024.1332890

**Published:** 2024-01-31

**Authors:** Stephan Ortiz, Sean J. Pittock, Achim Berthele, Michael Levy, Ichiro Nakashima, Celia Oreja-Guevara, Kerstin Allen, Yasmin Mashhoon, Becky Parks, Ho Jin Kim

**Affiliations:** ^1^Alexion, AstraZeneca Rare Disease, Boston, MA, United States; ^2^Department of Neurology and Center for Multiple Sclerosis and Autoimmune Neurology, Mayo Clinic, Rochester, MN, United States; ^3^Department of Neurology, School of Medicine, Technical University of Munich, Munich, Germany; ^4^Department of Neurology, Massachusetts General Hospital and Harvard Medical School, Boston, MA, United States; ^5^Division of Neurology, Tohoku Medical and Pharmaceutical University, Sendai, Japan; ^6^Department of Neurology, Hospital Clínico Universitario San Carlos, Instituto de Investigacion Sanitaria San Carlos (IdISSC), Madrid, Spain; ^7^Departamento de Medicina, Facultad de Medicina, Universidad Complutense de Madrid, Madrid, Spain; ^8^Department of Neurology, Research Institute and Hospital of National Cancer Center, Goyang, Republic of Korea

**Keywords:** autoimmune, complement, exposure-response analysis, neuromyelitis optica spectrum disorder, pharmacokinetics, pharmacodynamics, ravulizumab

## Abstract

**Objective:**

To assess the pharmacokinetics and pharmacodynamics of the long-acting terminal complement 5 (C5) inhibitor ravulizumab in adults with anti-aquaporin-4 antibody-positive (AQP4+) neuromyelitis optica spectrum disorder (NMOSD) in the phase 3, open-label CHAMPION-NMOSD trial (NCT04201262).

**Methods:**

Patients aged 18 years or older received a weight-based intravenous loading dose of ravulizumab (2,400–3,000 mg) on day 1, followed by weight-based maintenance doses (3,000–3,600 mg) on day 15 and once every 8 weeks thereafter. Pharmacokinetic assessments were maximum observed concentration (*C*_max_, assessed at the end of the infusion) and concentration at the end of the dosing interval (*C*_trough_, assessed before dosing) for ravulizumab. Pharmacodynamic assessment was time-matched observed free C5 concentration in serum up to 50 weeks.

**Results:**

The pharmacokinetic/pharmacodynamic analysis included 58 patients treated with ravulizumab. Serum ravulizumab concentrations at or above the therapeutic threshold (175 μg/mL) were achieved in all patients after administration of the first dose and maintained for 50 weeks. At week 50, the mean (standard deviation) *C*_max_ (*n* = 51) and *C*_trough_ (*n* = 52) were 1,887.6 (411.38) and 764.4 (217.68) μg/mL, respectively. Immediate and complete terminal complement inhibition (free C5 serum concentrations < 0.5 μg/mL) was achieved by the end of the first ravulizumab infusion and sustained throughout the treatment period. No treatment-emergent antibodies to ravulizumab were observed. No impact on ravulizumab pharmacokinetics was seen for age, sex, race, hematocrit, hemoglobin, markers of renal and liver impairment, or medications commonly used by patients with NMOSD. Body weight and BMI were significant covariates of ravulizumab pharmacokinetics.

**Conclusions:**

Serum ravulizumab concentrations were maintained above the therapeutic threshold in all patients through 50 weeks of treatment. Ravulizumab achieved immediate and complete terminal complement inhibition that was sustained throughout the treatment period in adults with AQP4+ NMOSD.

## 1 Introduction

Anti-aquaporin-4 antibody-positive (AQP4+) neuromyelitis optica spectrum disorder (NMOSD) is a rare, severe, disabling autoimmune inflammatory disease of the central nervous system (CNS) that predominantly affects the optic nerve, the brainstem and the spinal cord. NMOSD is characterized by unpredictable, severe relapses (attacks) that can cause irreversible neurological damage and disability (1–4).

The risk of NMOSD relapse persists even after long periods of remission (3). Effective and tolerable treatments that prevent relapses are required indefinitely (5, 6). In AQP4+ NMOSD, binding of serum aquaporin-4 immunoglobulin G (AQP4-IgG) to the aquaporin-4 water channel, which is highly expressed on astrocytic foot processes in the CNS, causes the activation of the complement cascade, the cleavage of complement component 5 (C5) into the potent proinflammatory anaphylatoxin, C5a, and C5b, which is a critical coordinator of the subsequent formation of the membrane attack complex (MAC) (7–10). Formation of C5a and C5b leads to astrocyte necrosis, which, in association with blood–brain barrier permeabilization, results in attacks that typically involve the optic nerve and spinal cord (8).

Eculizumab is a humanized monoclonal antibody that specifically binds to human C5, preventing the cleavage of C5 into C5a and C5b. In a phase 3 study that led to regulatory approval (PREVENT, ClinicalTrials.gov number NCT01892345), eculizumab was associated with a 94.2% relapse risk reduction compared with placebo in patients with AQP4+ NMOSD (11).

Ravulizumab is a second-generation antibody, developed from eculizumab, that retains high specificity for the same C5 epitope (12). Ravulizumab has four amino acid changes introduced into the eculizumab frame. These changes facilitate the dissociation of ravulizumab from C5 at endosomal pH and enhance neonatal Fc receptor-mediated recycling, resulting in extended elimination half-life (12) and corresponding increased duration of pharmacological activity (13). Pharmacological modeling supports an extended dosing interval of once every 8 weeks (6 doses per year) for ravulizumab compared with once every 2 weeks for eculizumab (26 doses per year). The longer dosing interval of ravulizumab is beneficial for patients in that fewer infusions, and therefore clinic visits that disrupt daily life and impact work productivity, are required to receive treatment with ravulizumab compared with eculizumab. In the primary treatment period of the CHAMPION-NMOSD trial (NCT04201262), no patients receiving ravulizumab (*n* = 58) had an adjudicated on-trial relapse, whereas 20 patients in the placebo group (*n* = 47) of the PREVENT trial, which was used as external comparator, experienced an adjudicated on-trial relapse (14). The long-term extension period of the CHAMPION-NMOSD trial is ongoing (estimated completion is July 2024). Ravulizumab has recently been approved in Europe, Brazil and Japan for the treatment of adult patients with AQP4+ NMOSD (15–17).

This analysis describes the pharmacokinetic and pharmacodynamic properties and immunogenicity of ravulizumab (weight-based maintenance dosing every 8 weeks) in adults with AQP4+ NMOSD using data collected in the primary treatment period of the CHAMPION-NMOSD study.

## 2 Methods

### 2.1 Study design and study population

**Figure 1:**
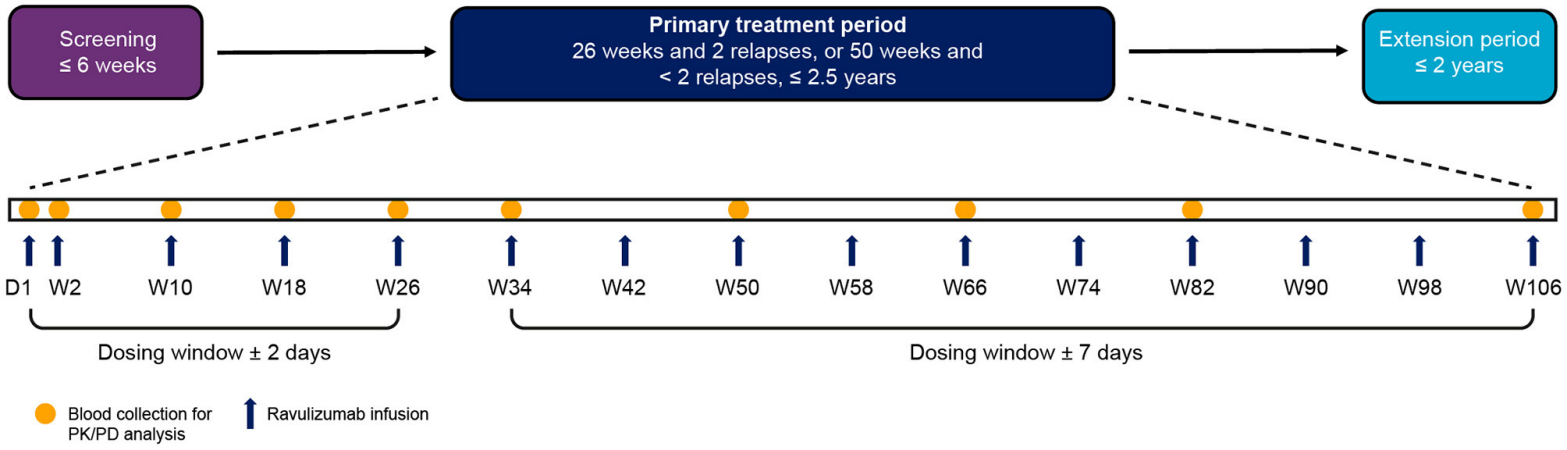
Study visit timeline. PK and PD analyses were performed on blood samples collected from patients during the primary treatment period. The end of the primary treatment period was to be triggered when two patients had an adjudicated on-trial relapse and all patients had completed, or discontinued before, 26 weeks on study. If two patients had not had an adjudicated on-trial relapse by the time all patients had completed, or discontinued before, 50 weeks on study, the end of the primary treatment period was to be triggered at that time. Because no patients had an adjudicated on-trial relapse during the study, the end of the primary treatment period was triggered when all patients had completed, or discontinued before, 50 weeks on study. Individual patients therefore spent different lengths of time in the primary treatment period depending on when they were enrolled. The maximum length of time a patient was enrolled in the primary treatment period was 117.7 weeks. D, day; PD, pharmacodynamic; PK, pharmacokinetic; W, week.

Details of the CHAMPION-NMOSD study design were described in detail in the primary publication (14). Briefly, the study comprised four periods: screening, primary treatment, long-term extension, and safety follow-up (Figure 1). The study population for this analysis included all 58 enrolled patients who received ravulizumab, of whom 56 completed the primary treatment period. Demographics and baseline characteristics of the study population are presented in Table 1.

**Table 1:** Demographic and baseline clinical characteristics.

**Demographic or characteristic**	**Ravulizumab (*n* = 58)**
**Age at first dose, years**	
Mean (SD)	47.4 (13.8)
Median (range)	46.0 (18.0–74.0)
**Age category**, ***n*** **(%)**	
< 45 years	25 (43.1)
≥ 45 years	33 (56.9)
**Age category**, ***n*** **(%)**	
18– < 65 years	51 (87.9)
≥ 65 years	7 (12.1)
**Sex**, ***n*** **(%)**	
Male	6 (10.3)
Female	52 (89.7)
**Race**, ***n*** **(%)**	
Asian	21 (36.2)
Black or African American	6 (10.3)
White	29 (50.0)
Unknown	2 (3.4)
**Japanese patient**, ***n*** **(%)**	
Yes	9 (15.5)
**Region**, ***n*** **(%)**	
Americas	21 (36.2)
Europe	17 (29.3)
Asia-Pacific	20 (34.5)
**Body weight, kg**	
Mean (SD)	69.9 (19.3)
Median (range)	63.8 (41.0–124.7)
**BMI, kg/m** ^2, a^	
Mean (SD)	26.7 (6.5)
Median (range)	25.7 (17.7–45.8)

^a^*n* = 56.

BMI, body mass index; SD, standard deviation.

### 2.2 Endpoints

The primary endpoint of CHAMPION-NMOSD was the time to first adjudicated on-trial relapse and associated relapse risk reduction. For this analysis, the pharmacokinetic endpoint was defined as the concentration of ravulizumab in serum at each study visit. The pharmacodynamic endpoint was the concentration of free C5 in serum at each study visit. The immunogenicity endpoint was the presence of anti-drug antibodies (ADAs).

### 2.3 Inclusion/exclusion criteria

Detailed inclusion and exclusion criteria are described in the primary publication (14). In brief, patients were eligible if they were aged 18 years or older and had a diagnosis of AQP4+ NMOSD according to the 2015 International Panel for NMO diagnosis diagnostic criteria, a history of at least one relapse in the 12 months before screening, and a score of 7.0 or less on the Expanded Disability Status Scale (3, 18).

### 2.4 Treatment

Patients received a weight-based intravenous loading dose of ravulizumab (2,400–3,000 mg) on day 1, then weight-based maintenance doses (3,000–3,600 mg) on day 15 and once every 8 weeks thereafter (Figure 1). The dosing window was ± 2 days from day 1 to week 26 (including day 15), and ± 7 days from week 34 onwards. The weight-based dosing regimen of ravulizumab can be found in Table 2.

**Table 2:** Body-weight-based dosing of ravulizumab.

**Body weight, kg^a^**	**Dose, mg**	
	**Loading**	**Maintenance**
40– < 60	2,400	3,000
60– < 100	2,700	3,300
≥ 100	3,000	3,600

^a^The dosing regimen was based on the last body weight measurement recorded during a study visit. This was commonly the current visit because body weight was measured before dose preparation on the day of the visit. If the study drug was prepared the night before a visit, the body weight measurement from the most recent prior study visit was used.

At the discretion of the investigator, patients were permitted to continue receiving supportive immunosuppressive maintenance therapy if they had established stable use before the start of the trial. Intravenous immunoglobulin G (IVIg), intravenous methylprednisolone, plasma exchange (PE), and plasmapheresis (PP) were permitted for the treatment of an on-trial physician-determined relapse, at the discretion of the treating physician. If IVIg, PE, or PP were administered, supplemental dosing of ravulizumab was provided to offset the decline in ravulizumab concentration associated with these therapies.

### 2.5 Sample collection

Given that no patient experienced an adjudicated on-trial relapse, the primary treatment period ended when all patients had completed at least 50 weeks of treatment or discontinued earlier in the study; therefore, patients may have received treatment with ravulizumab for more than 50 weeks, depending on their enrollment date.

Pharmacokinetic and pharmacodynamic parameters for ravulizumab were derived from individual serum concentration data for all patients who received at least one dose of ravulizumab and who had evaluable pharmacokinetic and pharmacodynamic data.

Blood samples for determination of serum drug concentrations and pharmacodynamic assessments were obtained before and after the administration of the study drug. Samples for baseline, trough, and post-dose pharmacokinetic and pharmacodynamic analyses were collected at the study visits indicated in Figure 1. Samples for baseline and trough analyses were collected in the 90 min before study drug infusion, whereas peak post-dose samples were collected in the 60 min after completion of study drug infusion.

Samples for analyses for the presence of ADAs were collected on day 1 (baseline), then before dosing at weeks 26 and 50, and then periodically at prespecified study visits every 24–32 weeks. Serum samples for ADA analyses were collected 5–90 min before the start of infusion of study drug from all patients. Additionally, serum samples were also collected at the final visit from patients who discontinued ravulizumab or who withdrew from the study.

Following collection, ADA, pharmacokinetic, and pharmacodynamic samples were stored upright for 30 min at room temperature to allow blood to clot. Samples were then centrifuged at 2–8°C for 10 min at 1,300 xg. Serum was then aliquoted into 2 mL cryovials, and frozen at −70°C (preferred) or −20°C and shipped on dry ice to a PPD laboratory (PPD Laboratories, Richmond, VA, USA) for testing.

### 2.6 Pharmacokinetic, pharmacodynamic, and immunogenicity endpoints

Prespecified pharmacokinetic endpoints were change in serum ravulizumab concentration over time, maximum observed ravulizumab serum concentration (*C*_max_), and serum concentration of ravulizumab at the end of the dosing interval (*C*_trough_). Pharmacodynamic effects of ravulizumab were evaluated by assessing the absolute values of free C5 in serum, as well as the numerical and percentage changes in free C5 levels from baseline over time. Complete inhibition of terminal complement activity was defined as a free C5 serum concentration below 0.5 μg/mL. The threshold of free C5 serum concentration of below 0.5 μg/mL has been previously established as representing complete terminal complement inhibition (19). The immunogenicity endpoint was defined as presence and titer of ADAs over the study duration.

#### 2.6.1 Exploratory analysis

Exploratory analysis included population pharmacokinetic evaluation of the impact of the following covariates on ravulizumab pharmacokinetics: age; race; sex; body weight; body mass index (BMI); hematocrit; hemoglobin; markers of renal function, namely creatine clearance and estimated glomerular filtration rate; markers of liver function, namely alanine aminotransferase, albumin, alkaline phosphatase, aspartate transaminase, and bilirubin; concomitant medication, namely antianemic preparations, antibacterials for systemic use, antihypertensives, antimycotics for systemic use, antithrombotic agents, corticosteroids for systemic use, and immunosuppressants; and immunogenicity.

### 2.7 Analyses

Pharmacokinetic, pharmacodynamic, and immunogenicity parameter analyses were carried out at an accredited PPD laboratory.

#### 2.7.1 Ravulizumab concentration

Total (bound and free) ravulizumab serum concentrations were determined using liquid chromatography with tandem mass spectrometry, with sensitivity [lower limit of quantification (LLOQ)] of 1.00 μg/mL and precision [coefficient of variation (CV%)] of up to 9.13%.

#### 2.7.2 Free C5 concentration

Free C5 concentration was measured using a Gyros-based fluorescence assay (Gyrolab^®^, Uppsala, Sweden), with sensitivity (LLOQ) of 0.0183 μg/mL and precision (CV%) of up to 4.64%.

#### 2.7.3 Immunogenicity

ADA concentration was semi-quantitatively measured using an electrochemiluminescence ligand-binding assay with sensitivity (LLOQ) of 80.7 and 49.2 ng/mL for the screening and confirmatory assays, respectively. Assay precision (CV%) was up to 16.1%. Neutralizing antibodies (NAbs) were semi-quantitatively measured using the Meso Scale Discovery Electrochemiluminescence assay (Meso Scale Diagnostics, Rockville, MD, USA), with a cutoff point of 24.4%, sensitivity of 0.443 μg/mL, and precision (CV%) of up to 12.5%.

### 2.8 Statistical analyses

Pharmacokinetic and pharmacodynamic analyses were performed on the pharmacokinetic/pharmacodynamic set, comprising all patients who received at least one dose of the study drug and who had at least one evaluable pharmacokinetic or pharmacodynamic result. Descriptive statistics for continuous variables were the number of patients, mean, standard deviation (SD), minimum, and range.

Descriptive statistics of ravulizumab concentration and pharmacodynamic endpoints are reported for each scheduled sampling time point for which data were available. The pharmacodynamic effects of ravulizumab were evaluated by assessing the absolute values of free C5, as well as the numerical and percentage changes in free C5 levels from baseline over time. The effects on the pharmacokinetic and pharmacodynamic profiles of ravulizumab of age, sex, race, body weight, BMI, hematocrit, hemoglobin, markers of renal and liver function, concomitant medication, and immunogenicity were analyzed by non-linear mixed-effects modeling NONMEM software, version 7.3 or higher (ICON Development Solutions, Hanover, MD, USA) on data collected up to week 50. The incidence of ADAs and NAbs and antibody titers were summarized using descriptive statistics.

## 3 Results

### 3.1 Patients

In total, 58 patients from 36 sites and 11 countries were enrolled in CHAMPION-NMOSD, and all received at least one dose of ravulizumab. Two patients who received ravulizumab discontinued treatment owing to adverse events before the end of the primary treatment period (14). Fifty-six of the 58 patients completed the primary treatment period. Patient demographics, clinical characteristics, efficacy, and safety from CHAMPION-NMOSD have been reported previously (14). Briefly, the median (range) treatment duration was 73.5 (13.7–117.7) weeks (*n* = 58). Of ravulizumab-treated patients, 52 patients (89.7%) were female, 29 (50.0%) were white, and 21 (36.2%) were Asian; there were nine patients (15.5%) of Japanese descent. Seven patients (12.1%) were aged 65 years or older. The mean (SD) and median (range) in body weight was 69.9 (19.34) and 63.8 (41.0–124.7) kg, respectively (Table 1). No doses of ravulizumab were missed by any patient during the primary treatment period.

### 3.2 Pharmacokinetics

**Figure 2:**
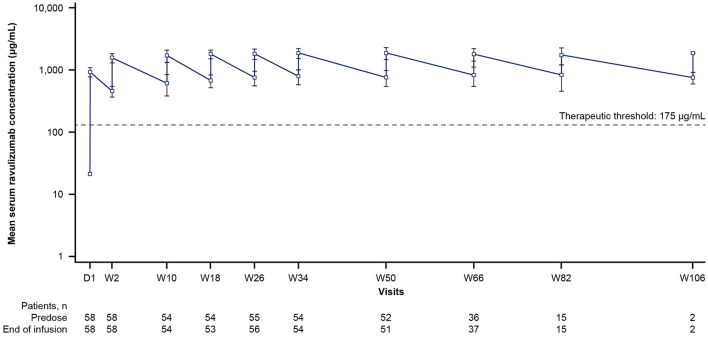
Mean ravulizumab serum concentration over time (pharmacokinetic/pharmacodynamic set). The *y*-axis is presented on a semi-log scale. The dotted line represents the therapeutic threshold of 175 μg/mL. Error bars represent SD. D, day; SD, standard deviation; W, week.

By the end of the first infusion, mean serum ravulizumab concentrations at or above the therapeutic threshold (175 μg/mL) were achieved in the 60 min after dosing and were maintained through week 50 in all patients (Figure 2). The body weight-based maintenance dosing (once every 8 weeks) maintained steady-state serum concentrations of ravulizumab above the therapeutic threshold for all patients. The mean (SD) and median *C*_max_ and *C*_trough_ of ravulizumab are presented in Table 3. At week 50, mean (SD) *C*_max_ was 1,887.6 (411.38) μg/mL (*n* = 51) and *C*_trough_ was 764.4 (217.68) μg/mL (*n* = 52).

**Table 3:** Ravulizumab pharmacokinetic parameters after maintenance dosing (pharmacokinetic/pharmacodynamic set).

	**All patients**	**Body weight 40– < 60 kg**	**Body weight 60– < 100 kg**	**Body weight ≥ 100 kg**
*C*_max_, μ**g/mL**	***n*** = **51**	***n*** = **21**	***n*** = **25**	***n*** = **5**
Mean (SD)	1,877.6 (411.4)	2,038.6 (338.4)	1,857.5 (410.9)	1,404.0 (334.6)
Median (range)	1,900.0 (607–3,080)	1,950.0 (1,630–3,080)	1,900.0 (607–2,460)	1,300.0 (1,070–1,890)
*C*_trough_, μ**g/mL**	***n*** = **52**	***n*** = **21**	***n*** = **26**	***n*** = **5**
Mean (SD)	764.4 (217.7)	856.8 (211.6)	741.2 (179.7)	497.4 (203.0)
Median (range)	770.0 (359–1,390)	793.0 (544–1,390)	719.0 (398–1,020)	399.0 (359–838)

The week 50 end-of-infusion sample was used for *C*_max_ and the week 50 predose sample was used for *C*_trough_. *C*_max_, maximum observed serum concentration; *C*_trough_, concentration at the end of the dosing interval; SD, standard deviation.

### 3.3 Population pharmacokinetics: covariate evaluation

Age, sex, race, hematocrit, hemoglobin, markers of renal and liver impairment, concomitant medications, and presence of ADAs did not have a significant impact on the pharmacokinetics of ravulizumab. Body weight and BMI were significant covariates of ravulizumab pharmacokinetics; however, these are accounted for by the weight-based dosing regimen.

### 3.4 Population pharmacokinetics: drug–drug interactions

Concomitant administration of medications, including anti-anemic preparations, antibacterials for systemic use, antihypertensives, antimycotics for systemic use, antithrombotic agents, corticosteroids for systemic use, and immunosuppressants had no statistically significant impact on the pharmacokinetics of ravulizumab.

### 3.5 Pharmacodynamics

**Figure 3:**
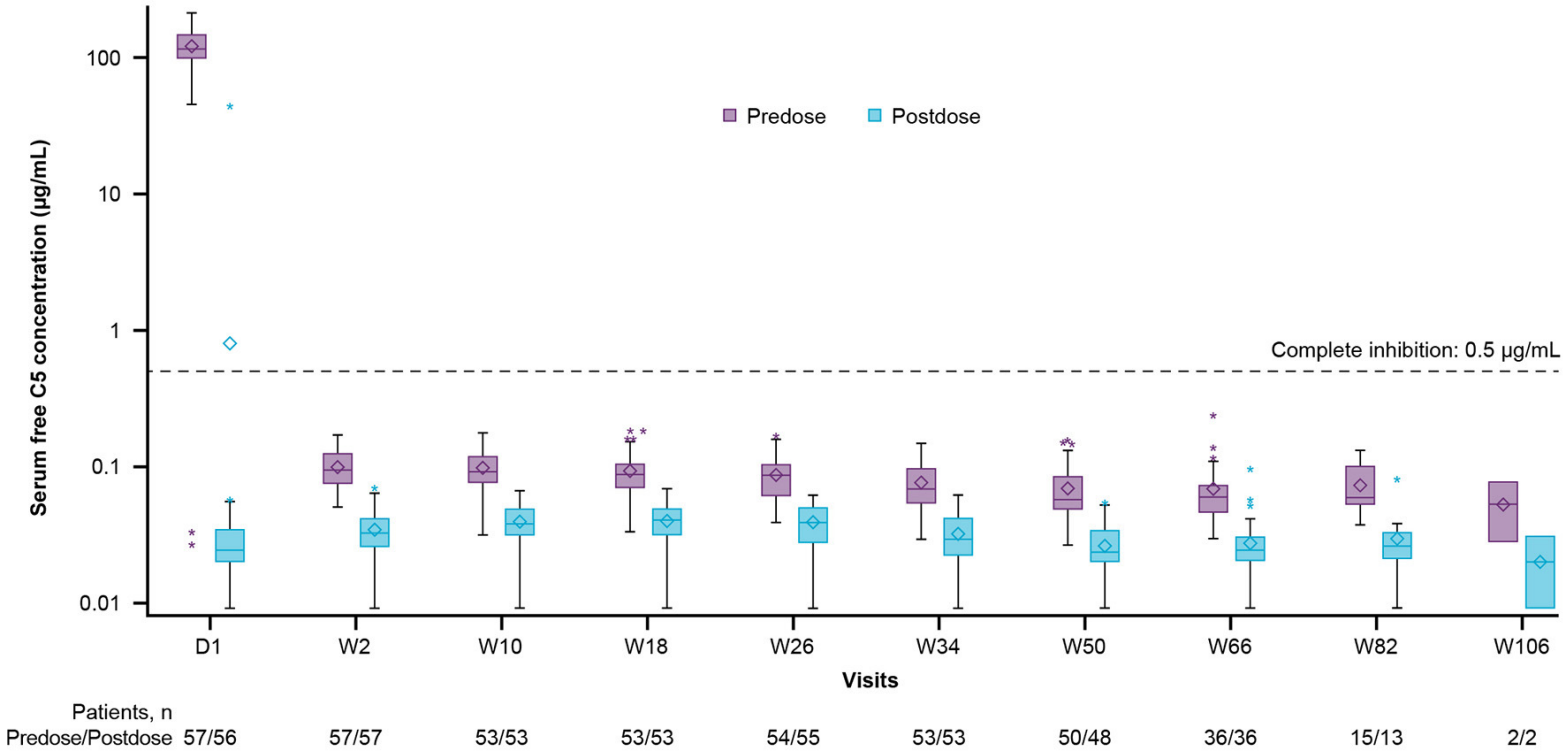
Free C5 serum concentration box-plots (pharmacokinetic/pharmacodynamic set). The *y*-axis is presented on a log scale. The horizontal line in the middle of each box indicates the median, the diamond indicates the mean, and the top and the bottom borders of the box mark the 75th and 25th percentiles, respectively. The whiskers represent the 1.5 IQRs of the lower quartile and upper quartile. Outliers are represented by an asterisk beyond the whiskers. The dashed horizontal line indicates a free C5 serum concentration of 0.5 μg/mL (values below which are defined as complete terminal complement inhibition). Data included in this figure are from scheduled visits only. C5, complement component 5; D, day; IQR, interquartile range; W, week.

At baseline, before the first dose of ravulizumab, the mean (SD) free C5 serum concentration was 120.10 (42.44) μg/mL [median (range), 115.00 (0.03–212.00) μg/mL], (*n* = 57). Immediate and complete terminal complement inhibition (free C5 serum concentrations < 0.5 μg/mL) was observed by the end of the first ravulizumab infusion and sustained throughout the primary treatment period (Figure 3). After the first dose (day 1), the median (range) free C5 serum concentration was 0.02 (0.01–44.10) μg/mL (*n* = 56); one patient had a free C5 serum concentration of above 0.5 μg/mL, most likely due to an undetermined assay issue because the time-matched pharmacokinetic data were as expected.

### 3.6 Immunogenicity

Five patients (8.6%) had ADA-positive samples at baseline, before treatment initiation. These patients were classified as having pre-existing immunoreactivity, defined as an ADA-positive response at baseline, with either all post-first-dose ADA results negative or < 4-fold over the baseline titer level. One of these patients had an ADA-positive sample at week 26 with low titer; however, their baseline ADA titer could not be determined. Based on the definition for pre-existing immunoreactivity, and the low titer, this patient was determined to have had pre-existing immunogenicity. No patients developed treatment-emergent ADAs. No positive NAb results were observed for any of the ravulizumab-treated patients. No impact of immunogenicity on ravulizumab pharmacokinetics or pharmacodynamics was observed in patients with ADAs.

### 3.7 Supplemental doses and drug–drug interactions

During the primary treatment period, two patients required supplemental infusions of ravulizumab after treatment for non-adjudicated, physician-determined relapses. One patient received PP interventions every other day over 9 days, for a total of five instances and received three supplemental ravulizumab doses: two doses of 1,500 mg and one dose of 3,000 mg. Another patient received one IVIg intervention and received a supplemental ravulizumab dose of 600 mg.

## 4 Discussion

In the CHAMPION-NMOSD trial, no patients had an adjudicated on-trial relapse in the primary treatment period. This analysis of pharmacokinetic, pharmacodynamic, and immunogenicity data from the primary treatment period supports the body-weight-based dosing regimen for intravenous ravulizumab in adults with AQP4+ NMOSD to achieve immediate, complete, and sustained inhibition of C5. This is the first study of the pharmacokinetics and pharmacodynamics of ravulizumab in adults with AQP4+ NMOSD.

After the first dose of ravulizumab, therapeutic serum concentrations of ravulizumab were achieved in all patients, and serum ravulizumab concentrations were maintained at or above the therapeutic threshold of 175 μg/mL with no unexpected pharmacokinetic findings throughout the treatment period. Therapeutic concentrations of ravulizumab resulted in immediate and complete terminal complement inhibition (free C5 serum concentrations of < 0.5 μg/mL) by the end of the first ravulizumab infusion and sustained throughout the treatment period in patients with AQP4+ NMOSD.

Pharmacokinetic and pharmacodynamic profiles similar to those reported here were observed in studies in patients with atypical hemolytic uremic syndrome, generalized myasthenia gravis, and paroxysmal nocturnal hemoglobinuria (13, 20, 21). The immediate, complete, and sustained suppression of C5 after ravulizumab administration may account for the adjudicated on-trial relapse risk reduction as observed in the CHAMPION-NMOSD trial (14). A previous analysis of the pharmacokinetics and pharmacodynamics of eculizumab reported that drug serum concentrations achieved after the first dose were sufficient to provide complete inhibition of terminal complement, which was sustained throughout the treatment period (22). The extended half-life of ravulizumab provides an alternative treatment option to other approved treatments for patients with AQP4+ NMOSD that offers a convenient 8-week dosing interval with a 7-day dosing window, without risking incomplete C5 inhibition and the subsequent increased risk of relapse. Given that NMOSD is a disease requiring ongoing treatment, the predictable 8-week ravulizumab dosing interval is expected to provide an additional benefit to patient quality of life by reducing the number of infusions and thereby clinic visits and associated impact on work productivity compared with eculizumab.

Pharmacometric analyses indicated that age, sex, race, hematocrit or hemoglobin levels, markers of renal and liver impairment, concomitant medications, or the presence of ADAs were not covariates for pharmacokinetics. Pharmacometric analyses of the impact of assorted drug classes on ravulizumab showed no apparent drug–drug interactions among those patients with available data. The impact of prior immunosuppressive therapy upon ravulizumab pharmacokinetics and pharmacodynamics was not analyzed. However, irrespective of previous exposure, all patients achieved serum ravulizumab concentrations above the therapeutic threshold and free C5 serum concentrations indicating complete terminal complement inhibition. In addition, ravulizumab is unlikely to be metabolized by cytochrome P450 or other enzymatic routes. No treatment-emergent immunogenicity was observed after ravulizumab treatment in patients with AQP4+ NMOSD. The absence of ADAs and NAbs suggests that the long-term efficacy and safety of ravulizumab may be expected to be maintained. Comparison between ethnic groups was restricted by the low proportion of Black or African American patients.

## 5 Conclusion

These pharmacokinetic and pharmacodynamic analyses support the weight-based dosing regimen to achieve immediate, complete, and sustained inhibition of terminal complement by ravulizumab for the prevention of relapses in adults with AQP4+ NMOSD.

## Data availability statement

Alexion will consider requests for disclosure of clinical study participant-level data provided that participant privacy is assured through methods like data de-identification, pseudonymization, or anonymization (as required by applicable law), and if such disclosure was included in the relevant study informed consent form or similar documentation. Qualified academic investigators may request participant-level clinical data and supporting documents (statistical analysis plan and protocol) pertaining to Alexion-sponsored studies. Further details regarding data availability and instructions for requesting information are available in the Alexion Clinical Trials Disclosure and Transparency Policy at http://alexion.com/our-research/research-and-development. Link to Data Request Form (https://alexion.com/contact-alexion/medical-information).

## Ethics statement

The studies involving humans were approved by the Institutional Review Boards at each participating institution. The trial was conducted in accordance with the provisions of the Declaration of Helsinki, the International Conference on Harmonisation guidelines for Good Clinical Practice, local legislation and institutional requirements, and applicable regulatory requirements. All patients provided written informed consent before participation.

## Author contributions

SO: Conceptualization, Formal analysis, Investigation, Supervision, Writing—original draft, Writing—review & editing. SJP: Writing—original draft, Writing—review & editing, data acquisition. AB: Writing—original draft, Writing—review & editing, data acquisition. ML: Writing—original draft, Writing—review & editing. IN: Writing—original draft, Writing—review & editing, data acquisition. CO-G: Writing—original draft, Writing—review & editing, data acquisition. KA: Data curation, Formal analysis, Methodology, Writing—original draft, Writing—review & editing. YM: Conceptualization, Methodology, Writing—original draft, Writing—review & editing. BP: Conceptualization, Methodology, Writing—original draft, Writing—review & editing. HJK: Writing—original draft, Writing—review & editing, data acquisition.
